# From injury to outcome: A mixed-methods study of animal-related injuries in a rural district of Tanzania

**DOI:** 10.1371/journal.pntd.0013494

**Published:** 2025-09-02

**Authors:** Gimbo Hyuha, Notikela Nyamle, Manase Kilonzi, Paul Malaba Makoye, Castory Sananga Chuwa, Beatrice Aiko, Nathanael Sirili

**Affiliations:** 1 Emergency Medicine Department, Muhimbili National Hospital, Dar es Salaam, Tanzania; 2 Emergency Medicine Department, Muhimbili University of Health and Allied Sciences, Dar es Salaam, Tanzania; 3 School of Public Health and Social Services, Muhimbili University of Health and Allied Sciences, Dar es Salaam, Tanzania; 4 School of Pharmacy, Muhimbili University of Health and Allied Science, Dar es Salaam, Tanzania; 5 Department of Health, Social Welfare and Nutrition services, Mkinga District Council, Tanga, Tanzania; Fundação de Medicina Tropical Doutor Heitor Vieira Dourado: Fundacao de Medicina Tropical Doutor Heitor Vieira Dourado, BRAZIL

## Abstract

**Background:**

Animal-related injuries remain a neglected public health issue in rural Tanzania, exacerbated by limited access to care and varied occupational exposures. While some studies have examined bite management in tertiary hospitals, little is known about the situation at the primary healthcare (PHC) level. This study explored clinical presentations, management and outcomes of animal-related injuries in a rural Tanzanian setting, using Mkinga District as a case study.

**Methods:**

A mixed-methods study was conducted in February 2024 across 29 PHC facilities in Mkinga, Tanzania. The quantitative component involved a retrospective audit of animal-related injury records from 2019 to 2023. The qualitative component comprised 10 interviews with facility in-charges to explore clinical decision-making, treatment approaches, and outcomes. Quantitative data were analyzed using SPSS; thematic analysis was applied to the qualitative transcript.

**Results:**

A total of 351 cases were documented. Symptom data were missing in over 70% of records, limiting clinical profiling. Among recorded cases, corticosteroids (55%) and antihistamines (53%) were commonly used, especially for insect and dog bites. Antibiotics were administered in insect stings (26%) and dog bites (23%), and analgesics were frequently used for insect bites. Antidotes were most common in dog (58%) and snake bites (26%). Of two cat bite cases, only one received antibiotics; neither received tetanus toxoid, despite the known infection risk. Qualitative findings highlighted three themes: clinical presentations, treatment modalities, and outcomes. Respondents described primary (e.g., bleeding, pain) and systemic (e.g., respiratory distress, neurological signs) symptoms. Treatment involved a mix of pharmacological, non-pharmacological, and traditional methods. While most patients recovered, some experienced complications or death.

**Conclusion:**

PHC facilities manage most animal-related injuries effectively, but gaps in documentation, guideline adherence, and referral systems remain. Strengthening provider training, improving resources, and engaging traditional healers may enhance timely care. Broader surveillance and community education are critical to reducing preventable harm.

## Author summary

Animal-related injuries, such as bites, stings, and scratches, are a serious but often neglected health issue in rural Tanzania. These injuries can cause infections, permanent disability, or death, especially when care is delayed or incomplete. Despite their importance, few studies have focused on this problem in sub-Saharan Africa, and even fewer in Tanzania. Most existing research centres on large urban hospitals, while most Tanzanians live in rural villages relying on small clinics as their first and often only point of care. This study examined how 29 rural clinics in northeastern Tanzania manage animal-related injuries. We reviewed four years of patient records and interviewed healthcare providers to understand patient presentations, treatments given, and outcomes. While most patients recovered, some suffered severe complications, often linked to delayed care or inappropriate treatment. Treatments ranged from modern medical care (antibiotics, vaccines, antivenoms) to traditional remedies. We identified that animal-related injuries present with a wide range of symptoms and signs, but there are major gaps in treatment and adherence to national guidelines. Our findings emphasize the urgent need to improve healthcare worker training, strengthen primary care systems, ensure reliable access to essential medicines, and engage communities in education and prevention. These steps are crucial to reducing preventable harm from animal injuries in rural, resource-limited settings like Mkinga.

## Introduction

Animal-related injuries are a neglected public health issue, particularly in tropical countries such as Tanzania [1–5]. These injuries vary widely in type and severity, including bites, stings, stomping, lacerations, crushing injuries, goring, being bucked off, falls caused by animals, pecks, and scratches. Among these, animal bites are often considered contaminated and carry a high risk of infection and envenomation [1]. According to the World Health Organization (WHO), animal bites can be categorized into three main groups: common bites (e.g., from snakes and dogs), bites from other vertebrates (including cats, rodents, monkeys, and wildlife), and bites from invertebrates (such as insects, scorpions, jellyfish, and sea urchins) [6]. These bites can further be classified as venomous or non-venomous, possessing the potential to transmit venom, toxins, and pathogens, contributing to significant morbidity and mortality worldwide, with the highest burden observed in Africa and Asia [6].

The clinical presentation and management of animal-related injuries vary depending on the type of animal involved, resulting in a broad range of outcomes and complications [1,2,5,7,8]. Symptoms range from minor discomfort to severe manifestations like loss of consciousness and anaphylaxis [2]. While bruises are the most common form of injury, more severe cases may involve crush injuries or even amputations [2]. Regardless of severity and presentation, prompt medical intervention is essential and can be lifesaving. Initial treatment should focus on symptom relief and supportive care, with an emphasis on infection prevention, tetanus immunisation, and appropriate wound management, including the use of antibiotics. This is especially important because polymicrobial infections are commonly associated with most injuries, especially bites. Although many animal-related injuries can be treated conservatively, extensive wounds may require surgical debridement and reconstructive procedures [2,9].

While there is no “one-size-fits-all” approach to managing animal injuries, understanding the typical clinical presentations and treatment strategies is crucial for healthcare providers (HCPs). This knowledge enables HCPs to deliver timely, effective care and improve patient outcomes. In resource-limited settings, supportive care remains a critical component despite ongoing efforts to expand access to advanced treatments and improve patient education [9]. Simple interventions such as removing the victim’s clothes and jewellery, providing resuscitation fluids and supportive oxygen, wound care, and administering antibiotics targeting common pathogens based on local animals and geography can be both cost-effective and efficient for treating minor wounds, especially in rural areas [8,9].

The outcome of animal injuries after care can range from survival to surviving with lifelong disability, or, in severe cases, death, either pre-hospital or in the hospital [3,6,7]. In limited-resource countries, the outcome of animal-related injuries is often poor, and this is frequently worsened by the fact that patients present to health facilities very late [4,5]. The reasons for the delayed presentation vary, ranging from the lack of prehospital care in many areas, financial constraints, limited access to effective ambulance services, a reliance on untested traditional medicine, and a general lack of community awareness regarding immediate interventions that could be life-saving [1,10–12].

In Tanzania, animal-related injuries are most frequently reported among young adult males, a key demographic group in terms of productivity [1]. A preliminary analysis conducted by the authors in Mkinga District, a rural area in northeastern Tanzania, found approximately 7 out of every 10,000 health facility visits were attributed to animal-related injuries. These data likely underestimate the true burden, as they include only cases that were formally reported. While international guidelines for bite management exist [8,9], clinical practice in Tanzania is primarily guided by the national Standard Treatment Guidelines and Essential Medicines List *(STG/NEMLIT)* [13,14] developed by the Ministry of Health in collaboration with stakeholders. These guidelines provide clinical presentation and outline both pharmacological and non-pharmacological management strategies.

Although the clinical management and outcomes of animal-related injuries have been documented in Tanzanian tertiary hospitals [1,4], there is a notable gap in the literature regarding such injuries in rural districts and at the primary healthcare level, where resources are constrained and access to advanced care is limited. This study aims to address that gap by using Mkinga, a district with diverse socio-economic activities, as a case study to examine the clinical presentations, treatment strategies, and outcomes of animal-related injuries in a rural Tanzanian context. We hypothesized that the clinical management and outcomes of animal-related injuries at PHC facilities in rural Tanzania are adversely affected by limited resources, inconsistent adherence to clinical guidelines, and the diverse nature of animal exposures. Specifically, we expected to find substantial variation in treatment practices, incomplete documentation, and preventable complications due to delays in care.. Unlike existing studies, this research focuses specifically on a primary health facility and the rural population it serves, offering a unique perspective that has not been adequately explored. The findings are intended to inform the development of locally relevant and context-appropriate management protocols for similar resource-limited settings. Ultimately, this study seeks to reduce the burden of animal-related injuries and to enhance awareness of effective treatment strategies and health system responses in Tanzania and comparable regions.

## Methods

### Study design

This mixed-methods study was conducted in February 2024. The quantitative component involved a retrospective audit of all documented cases of animal-related injuries recorded across 29 primary healthcare (PHC) facilities in Mkinga District from 2019 to 2023. The qualitative component aimed to explore clinical presentations, treatment approaches, and patient outcomes through in-depth interviews with healthcare providers (HCPs), all of whom also served as facility in-charges. This study is nested within a broader investigation that was designed to examine the incidence, clinical presentation, management practices, and challenges related to the availability and affordability of medications and supplies for treating animal-related bites. However, the present paper focuses specifically on findings related to clinical presentation, management strategies, and treatment outcomes.

### Study setting and context

#### Overview of the Tanzanian Health System.

**Fig 1:**
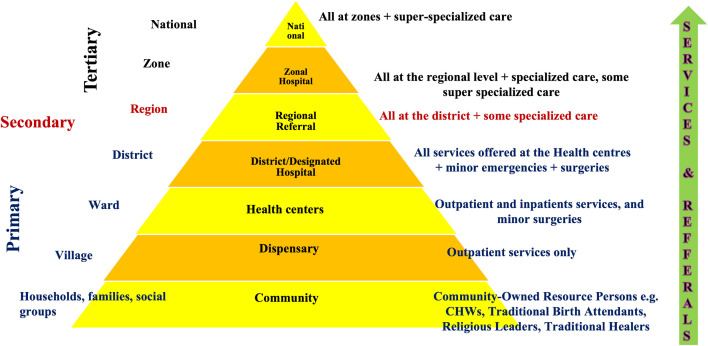
Tanzanian health system. This figure presents a simplified schematic of the hierarchical structure of the Tanzanian health system, illustrating the flow of service delivery and referrals across levels. At the base, community-level care is provided by community-owned resource persons like CHW. This is followed by the primary level, which includes dispensaries, health centres and district hospitals offering basic outpatient, maternal, child, and minor emergency services. Above them, regional referral hospitals deliver more advanced diagnostics, inpatient, and surgical services while overseeing district hospitals. At the top, zonal referral and national hospitals provide specialized tertiary care, education, and research. Arrows in the figure depict referral and supervisory pathways. The schematic was adapted and redrawn by the authors from the official Tanzanian Ministry of Health structure.

Tanzania, with a population of approximately 61.7 million (2022 census), is divided into 30 administrative regions: 25 on the mainland and 5 in Zanzibar Island. These regions are further subdivided into districts, divisions, wards, and villages. Approximately 65.1% of Tanzanians reside in rural areas [15,16]. The Tanzanian health system follows a pyramidal structure: Primary care is delivered at the village/community level through dispensaries and health centres. Secondary care is provided at regional hospitals, and Tertiary care is offered at national and zonal referral hospitals. Referral follows a bottom-up structure, with increasing availability of medication, other resources and expertise at higher levels of care (**Fig 1**).

#### Study area.

**Fig 2:**
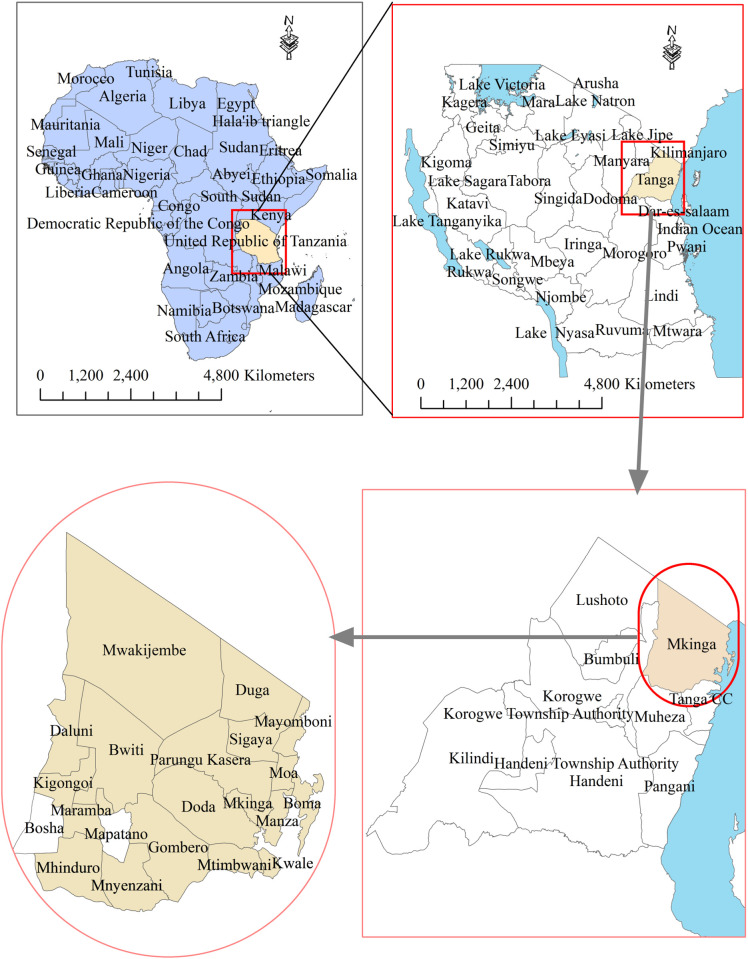
Map of Mkinga District, Tanga Region, Tanzania. This figure illustrates the geographic location of Mkinga District within Tanga Region in northeastern Tanzania. It highlights district boundaries, key geographical features such as water bodies, and the spatial distribution of districts for reference. The map was generated using ArcGIS software, version 10.7.1 (https://www.esri.com/software/arcgis). Administrative boundary shapefiles were sourced from Natural Earth (https://www.naturalearthdata.com), which are in the public domain. All map annotations and geospatial overlays were added by the authors. No proprietary or copyrighted basemaps were employed.

This study was conducted in PHC facilities in Mkinga District, located in the Tanga Region of northeastern Tanzania (**Fig 2**). Mkinga is a rural district with a population of 146,802 (2022 census), nearly equally divided between males and females. Covering 2,712 km^2^ along the Indian Ocean, the district comprises 42 operational PHC facilities, providing a practical framework for assessing community-level responses to animal bite incidents. The region experiences a tropical climate with high humidity and two rainy seasons: the long rains (*masika*) from mid-March to May, and the short rains (*vuli*) from November to mid-January. Coastal areas are typically hot and humid, while the inland plateau is cooler and drier during the dry season from May to October. Mkinga’s economy is based on sisal plantations, small-scale farming, fishing, livestock keeping, and petty trade—livelihoods that frequently expose residents to animal bite risks. The district was selected for this study due to a previously identified high burden of animal bites [17–19].

### Study participant and sample size

i ***Quantitative Component***: A list of PHC facilities was obtained from the District Medical Officer. Sites were selected based on geographic representation and the completeness of their patient records to ensure both spatial coverage and data quality. Inclusion criteria required facilities to have been continuously operational for four years (2019 -2023) with access to patient documentation. We naturally excluded those that were operational for less than 4 years and those with incomplete or inaccessible patient records, either due to lack of documentation in the system or system failure during the time of study (n=13). Based on this criterion, 29 out of 42 facilities were eligible and included in the study. These comprised 3 health centres and 26 dispensaries.

In terms of criteria used for cases, at each facility, we included cases were documented between 2019 and 2023, cases with clear diagnoses explicitly linked to animal-related injuries (e.g., dog bite, snake bite, insect sting) and lastly cases with sufficient information to extract treatment and/or outcome data. Naturally, we exclude cases with unclear diagnoses or non-animal-related injuries.

### Potential bias

*Information Bias, i.e., a mis-classification bias* due to these missing clinical presentation data, which may have caused us to overestimate or underestimate disease severity. Another potential bias is *selection bias* since those cases with missing data on clinical presentation may have differed systematically from those with complete data in terms of disease severity or management. Consequently, our ability to fully characterize the clinical spectrum and find associations between presentation and patient outcomes was limited.

ii ***Qualitative Component:*** To complement and contextualize the quantitative findings, 10 in-depth interviews (IDIs) were conducted with facility in-charges. The facility in charge was used due to the context that their key role is an oversight for clinical decision making and record keeping in a health facility, as per the Tanzania guideline. This included all three health centers and seven dispensaries, which were selected through simple random sampling. Interviews continued until thematic saturation was achieved.

### Variables, data source and data collection

i ***Outcomes***: The primary outcome of the study was the clinical presentation and management of animal and insect bites in rural Tanzania. A secondary outcome was the reported treatment outcomes associated with these cases.
ii ***Training***: A two-day training session was conducted for all research assistants (RAs), focusing on the use of qualitative checklists, interview techniques, and the structured case report form (CRF). All RAs were clinicians or nurses with previous experience in qualitative research and using the physical Hospital Information Management System (pHIMS). They also received study-specific training to ensure uniform and high-quality data collection across sites.
iii ***Quantitative Data Collection*:** Data were collected using a structured CRF. At each selected facility, records of animal-related injuries from 2019 to 2023 were retrieved from the pHIMS. Data collection was conducted by trained RAs with clinical backgrounds, ensuring familiarity with medical terminology and documentation.
iv ***Qualitative Data Collection***: An interview guide was developed based on the research team’s expertise in emergency care, a review of relevant literature, and established qualitative research principles (see S1 File). The guide was part of a broader tool designed to explore healthcare providers’ perceptions of incidence, clinical presentation, management practices, and challenges related to the availability and affordability of medications and supplies for treating animal-related bites. The guide was initially written in English and translated into Swahili for field use. In-depth interviews were conducted by three investigators in private office spaces within the facilities. Each session lasted between 30 and 60 minutes. Informed consent was obtained from all participants, and interviews were audio-recorded to ensure data accuracy. Following the first three interviews, the research team reviewed the process and made minor revisions to the guide to improve clarity and depth in the remaining interviews.

### Data analysis

#### Quantitative.

Data were entered into an MS Excel spreadsheet (2021) and then imported into SPSS software version 25 (IBM Corp., Armonk, NY, USA) for cleaning and analysis. Initial data cleaning included validation of code consistency, removal of duplicates, and screening for missing or/and out-of-range values. Since the study focused on categorical outcomes (i.e., treatment modalities, patient outcomes), all data were summarized as frequencies and percentages, with both numerators and denominators reported to ensure transparency. As this was a descriptive, exploratory audit with a limited sample size, no inferential statistical tests were performed due to insufficient statistical power. Nevertheless, all categories are fully reported with exact values to support reproducibility and to provide a detailed view of observed patterns.

#### Missing data handling.

Missing data were addressed through complete-case analysis; records missing documentation for key variables were excluded from the analysis. Symptom -level data were missing in over 70% of cases and thus excluded from formal analysis due to unreliability and inconsistent documentation. Furthermore, we noted that some recorded “symptoms” were actually diagnoses or reasons for visit (e.g., “came for rabies vaccine,” “dog bite”) rather than clinical signs, limiting meaningful symptom-level analysis.

We confirmed missing data as random by comparing excluded and included records by facility type and referral status using Chi-square tests, which showed no significant differences (p > 0.10), suggesting data were likely missing at random (MAR).

The sampling framework and transparent handling of missing data align with STROBE guidelines [20], which mandate clear reporting of selection methods, the extent of missing data, and how it is addressed in observational studies. *All our findings, including exact counts and percentages,* are available in S2 File to enable external verification and replication.

#### Qualitative.

Thematic analysis, as described by Braun and Clarke, was used [21]. All interviews were verbatim transcribed, and analysis was carried out in Swahili to preserve the original meaning. Only the abstracted codes and quotes were translated into English for presentation. A preliminary codebook was developed based on the study objectives and interview guide. Researchers familiarized themselves with transcripts through repeated reading. Two pairs of investigators independently coded an initial transcript, followed by a consensus meeting to refine the codebook and resolve discrepancies in coding. After agreement was reached, the remaining transcripts were coded independently. Codes were grouped into sub-themes and subsequently into broader themes. Key findings were supported with illustrative quotes.

### Ethical statement

Ethical clearance was secured from the Muhimbili University of Health and Allied Sciences - Research and Ethics Committee (*ref. number: MUHAS-REC-07-2023-1813*). Permissions were obtained from pertinent governmental bodies in Tanzania (the ministry and local governments), and participants provided written informed consent before interviews. The study adhered to the ethical principles outlined in the Declaration of Helsinki throughout the recruitment and data collection processes. All collected data was securely stored and accessible only to the principal investigator and authorized research team members.

## Results

### Quantitative

Between 2019 and 2023, a total of 351 animal-related injury cases were documented across 29 primary healthcare (PHC) facilities in Mkinga District, with 276 cases (78%) seen at dispensary-level facilities. Quantitative analysis focused on: treatment administered, patient disposition, and treatment outcomes.

### Treatment administered

**Fig 3:**
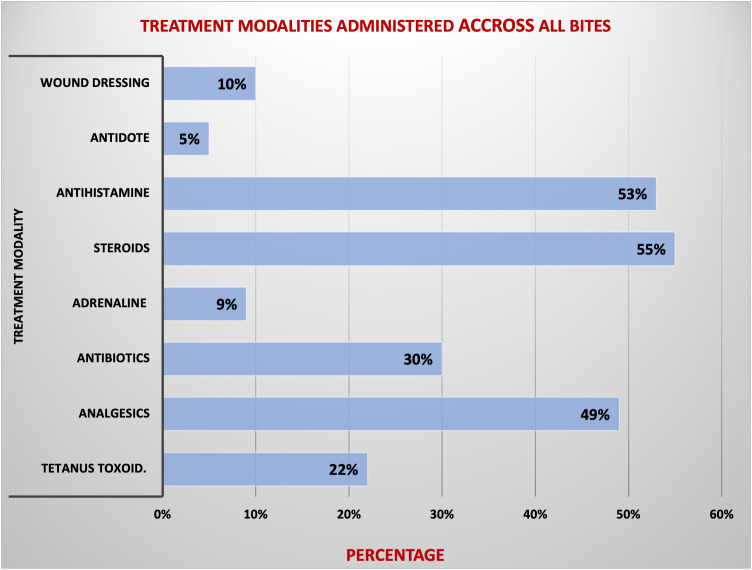
Treatment modalities administered across all bites (N = 351). Bar chart showing the distribution of eight treatments—steroids, antihistamines, antibiotics, analgesics, antidotes (antivenom or rabies PEP), wound dressing, tetanus toxoid, adrenaline- across all animal-related injury categories in 351 cases from Mkinga District selected facilities (2019–2023). Each bar is labeled with the percentage (%) of cases in which the treatment was administered. The figure highlights that *steroids and antihistamines* were the most frequently used treatment modalities.

**Fig 4:**
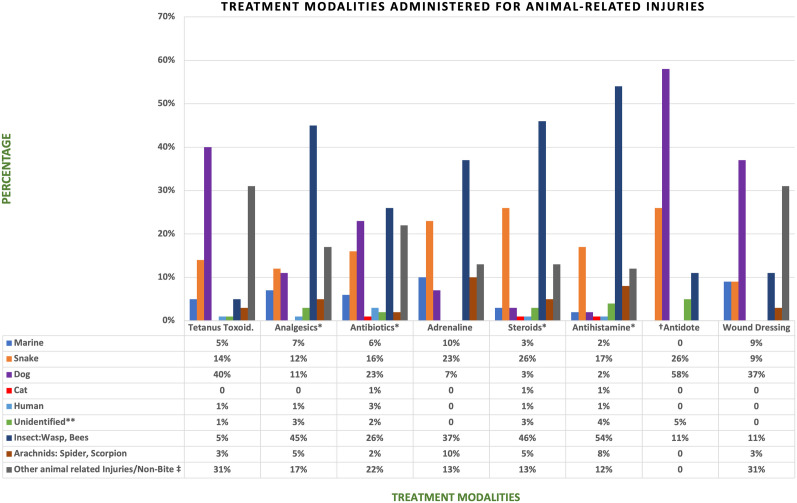
Treatment modalities administered for animal-related injuries. Each chart represents the percentage of patients within each treatment group who received that treatment for a given bite/injury type. Treatment modalities include tetanus toxoid (N = 78), analgesics (N = 172), antibiotics (N = 105), adrenaline (N = 30), steroids (N = 192), antihistamines (N = 187), antidotes (N = 19), and wound dressing (N = 35). **†Antidote** includes administration of antivenom or anti-rabies vaccines/PEP**. ‡non-bite injuries** include scratches, stomping, lacerations, or bruises from animal encounters. **Unidentified bites are for patients presenting with bite marks, but unsure of the animal that caused them./Could not identify. *Values have been rounded off totals may not sum to 100% but 101.

Eight distinct treatment modalities were identified, with the most frequently administered treatments being steroids (192/351, 55%) and antihistamines (187/351, 53%), predominantly used in insect bite and sting cases. Wound dressing was common in dog bites and non-bite injuries, which included animal-related trauma such as scratches, lacerations, bruises, and stomping. Antibiotics were commonly given in insect stings (26%) and dog bites (23%). Analgesics were mainly provided for insect stings (45%). Antidotes were most frequently administered in dog bites (58%) and snake bites (26%). Among two cat bite cases, only one received antibiotics, and neither was given tetanus toxoid despite the known infection risk. Treatment overlap across injury types likely reflects providers’ clinical judgment based on resource availability and case severity (**Figs 3** and **4**).

### Disposition and outcomes

**Fig 5:**
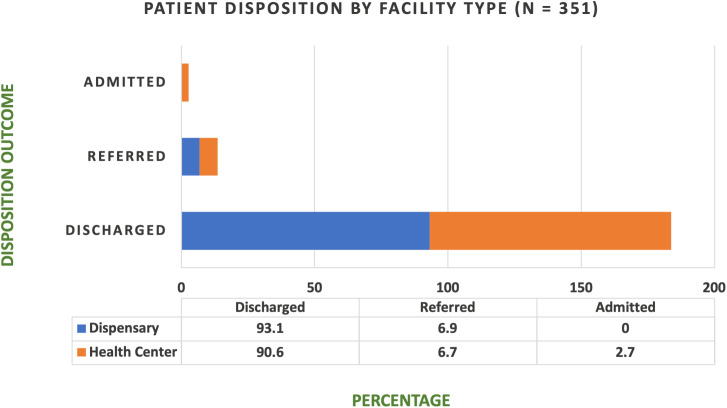
Patient disposition by facility type (n = 351). This stacked bar chart shows the disposition of patients with animal-related injuries across two primary healthcare facility types in Mkinga District: dispensaries and health centers. Disposition outcomes include discharged, referred, and admitted. As per Tanzanian health policy, dispensaries did not admit any patients, reflecting their restriction from providing inpatient services. The figure highlights differences in referral and admission patterns between facility types.

No in-facility deaths were reported during the study period. The majority of patients were treated and discharged from the primary facilities. No patients were admitted at dispensary-level facilities, in accordance with government regulations prohibiting inpatient care at that level **(see Fig 5).** Fewer than 10% of patients were referred to higher-level facilities. Chat reviews indicated that referrals were primarily for cases requiring rabies vaccination, antivenom administration, or surgical intervention.

### Qualitative

#### Respondents’ general characteristics.

A total of 10 in-depth interview (IDI) participants were included, comprising six males and four females. The sample consisted of three enrolled nurses (certificate holders), two nurse assistants (diploma holders), and five clinical officers (holders of a diploma in clinical medicine). Participants reported between two and ten years of clinical experience.

### Findings

Thematic analysis yielded three overarching themes: clinical presentations, treatment modalities, and treatment outcomes (Table 1).

**Table 1:** Clinical presentation, Management and Outcome of Animal Related Injuries in Mkinga District, Tanga, Tanzania.

ABSTRACTED CODES	SUBTHEMES	THEMES
• Pain • Bleeding • Swelling • Loss of Consciousness • Broken Arms and legs	• **Primary symptoms**	**COMMONLY ENCOUNTERED VICTIMS’ CLINICAL PRESENTATION**
• Difficulty in Breathing • Vomiting • Rabies Barking • Eye Protruding • Gangrene due to bite • Frothing	• **Systemic symptoms**	
• Bleeding control • Wound dressing • Use of icepack • Use of water and soap • IV access	**Non-pharmacological interventions**	**ADOPTED TREATMENT MODALITIES**
• Use of steroids • Use of anti-rabies • Use of antivenom • Use of antihistamines • Use of antibiotics • Use of antipains • Use of adrenalines • Use of antidiuretics/furosemide	**Pharmacological Interventions**	
• Use of traditional herbs • Type a rope above injury site, • Use of traditional snake stone, • Apply ash to wound, • Scarification of bite site, • Apply coin on bite site	**Traditional interventions**	
• Improve after management • Improve during follow up • Some are discharged	**Recovered Without Complications**	**ENCOUNTERED TREATMENT OUTCOMES**
• Disabled from bite • Has permanent scars	**Recovered with Complications**	
• Death at home • Death from bites	**Death**

1 **Common clinical presentations of victims**

Respondents described a broad spectrum of symptoms among bite victims, categorised into primary and systemic manifestations.

a) ***Primary symptoms***:

These included pain at the bite site, bleeding, swelling, and visible injuries such as fractures or lacerations.

*“…We receive clients who have been attacked by animals like crocodiles, and we’ve received many cases. Some people end up with severed arms, while others have broken arms or legs.”* (Participant 1)

**And**

*“...There was one case where he was bleeding so much, it would scare you.”* (Participant 2)

**And**

*“...He was gathering firewood and got bitten by that insect. He came here complaining of very severe pain.”* (Participant 7)

b) ***Systemic symptoms:***

Respondents reported neurological signs (e.g., frothing, “rabies barking”, eye rolling), respiratory distress, and signs of infection or inflammation (e.g., gangrene)

*“Or as usual, they didn’t say he had been bitten by a dog. Later, when they returned home, his condition began to change, and he started showing signs similar to those of a dog—barking like a dog, rolling his eyes* (Participant 3*).*

**And**

*‘Another person may come, and you can clearly see that they are having difficulty in breathing, so we have to provide them with medication that will help at least reduce the symptoms, even if it’s for an allergy, so that they can find some relief”* (Participant 6)

**And**

*The first one was experiencing dryness and foaming white at the mouth, like someone with poison in the bloodstream.” (Participant 1)*

2 **Treatment modalities**

Respondents described various treatment approaches, classified into non-pharmacological, pharmacological, and traditional interventions.

a) ***Non-pharmacological interventions***

Responders reported basic wound management practices, such as cleaning the wound with clean water and soap and applying pressure to stop bleeding.

*“We manage those cases with first aid, such as controlling bleeding, stitching, and putting in absorbable stitches, and then we refer the people to a referral hospital”* (Participant 5)

**And**

*When someone is bitten, you find that the area is swollen; we apply something like ice so that the swelling does not continue to increase. It’s one of the management strategies we use….I will clean the area where the bite occurred with clean water and soap. After that, I will check to see if there is anything left, such as teeth. If something remains, that’s when I will act* (Participant 9).

b) ***Pharmacological Interventions***

Respondents mentioned the use of several medications, including Steroids (e.g., prednisolone, dexamethasone) and Antihistamines (e.g., cetirizine). In specific cases, antivenoms were administered for snake bites, and rabies vaccines were given following dog bites. Unexpectedly, one respondent reported the use of an antidiuretic (furosemide) for certain animal bite cases.

*“Most of the time we use adrenaline, we use hydrocortisone, we use piriton, and prednisone; those are the medications that we often use”* (Participant 3)

**And**

“*After cleaning the wound, we start the patient on injections, tetanus, for example, you see*” (Participant 4)

**And**

*“For people bitten by dogs, we provide a protective vaccine—anti-rabies, to protect them from rabies.”* (Participant 10)

**And**

*“I’ll give them some other medication; tablets we call furosemide tabs. I’ll give 40 milligrams,…. The purpose of giving this is that it’s a diuretic drug…., the diuretics I’ve given will eventually make them feel the urge to urinate. The goal of urinating is also another way to help flush out the toxins from the body. After that, they’ll continue to rest as I monitor how the patient is progressing”* (Participant 9)

c) ***Traditional interventions***

Respondents observed that communities often rely on traditional remedies, including traditional snake stones, coins and herbal concussions. These interventions were widely used either before arriving at the facility or alongside medical treatments.

*“People from this area usually go to traditional healers first; by the time they come here, they’ve often already been treated with herbal medicine”* (Participant 3)

**And**

*“They began traditional treatment, saying the area should be cut (scarified) and a snake stone applied. It is believed that this helps reduce the effect if the bite is venomous”* (Participant 10)

**And**

*“They believe that the coin can draw out the venom from the body”* (Participant 5)

3 **Treatment outcomes**

Outcomes were grouped into three categories: **recovered**, **recovered with complications**, and **death**, reflecting the range of clinical courses observed.

a) ***Recovered without complications***

Most respondents indicated that the majority of animal bite victims treated at their facilities recovered well and were discharged without complications.

*“Since the service began, we have not recorded any deaths from snakebites, wasp stings, or marine envenomation. Our clients appear to recover well, and we’re grateful for that”* (Participant 6)

b) ***Recovered with complications***

Some respondents stated that victims developed long-term effects, with a few requiring amputations of an affected limb.

*There was a case where a patient’s foot had become severely necrotic following a bite from a kipili (a locally known viper species with highly potent venom)* (Participant 8).

c) ***Death***

Although rare, a few respondents reported witnessing deaths among animal bite victims. One facility recounted a case where a child died of rabies symptoms despite completing the vaccination series. These deaths typically occurred after referral to higher-level facilities (district or regional hospitals) or at home.

*A seven-year-old child came in after a dog bite and completed the full course of rabies vaccination. But about a month later, he returned with symptoms of rabies. We referred him to the regional hospital, but unfortunately, he passed away.”* (Participant 10)

## Discussion

We examined the clinical presentations, management strategies, and outcomes of animal-related injuries across 29 primary healthcare (PHC) facilities using four years of retrospective facility data, complemented by qualitative interviews with facility in-charges. We found that most patients presented with localised symptoms such as pain and swelling, and systemic manifestations like neurological signs, respiratory distress, and gangrene. Management approaches included non-pharmacological measures like wound cleaning and bleeding control, and pharmacological treatments such as corticosteroids and antihistamines, especially in insect bite cases. Antivenom and rabies post-exposure prophylaxis were administered when available, consistent with global best practices [1,2,6,7,9,22]. These findings are consistent with the global literature that classifies animal bites into minor and severe forms depending on exposure and animal type [1,2]. On the contrary, the existence of a high percentage of undocumented symptoms as revealed by our study is against the standard practice that requires systematic triage and injury classification to be part of the routine practice [8].

Although traditional beliefs and practices remain deeply embedded in the community, there is insufficient scientific evidence to support remedies such as snake stones, the application of ash or herbs, and scarification. A review by Beasley et al., examining 50 articles on traditional remedies for rabies and dog bites, including randomized controlled trials, found only one remedy that could be considered potentially useful for rabies treatment [10]. These findings align with concerns from our interviews, where delayed presentation to formal care was often linked to reliance on such traditional practices, potentially contributing to preventable complications, worse prognoses or death [10,11]. Our findings are consistent with other studies, which show that over 50% of rural patients in various African countries initially consult traditional healers [10,23]. While there is growing support for community health education, a gap remains in the engagement of informal providers in emergency contexts. The engagement of traditional healers can serve as an important link between cultural beliefs and evidence-based care in rural Tanzania and similar LMICs.

While other Tanzanian studies have reported permanent deformities, limb loss, and infections resulting from animal bites [1,2], this study found that most patients in Mkinga were discharged after initial management at primary health facilities, with few complications. This could be due to earlier presentation and faster management at the primary care level. In contrast, studies such as *Gilyoma’s,* conducted at a tertiary hospital, captured more severe or referred cases that could not be adequately managed at lower levels and required specialist care [1]. However, clinician interviews in our study reported deaths either before arrival at a facility or after referral, which are often linked to poor infrastructure, lack of emergency services, and long distances to care. These findings highlight the critical need to strengthen community-based emergency response systems, including transport and referral pathways.

It is also important to consider how the geography and socioeconomic activities in Mkinga, such as fishing in the Indian Ocean, working in large sisal plantations, and small-scale livestock farming, place residents at unique risk of various animal-related injuries. Clinical presentations differed depending on the type of exposure. For instance, insect bites typically resulted in pain and localised swelling, while crocodile attacks among fishermen led to severe trauma, including fractures and bleeding. Snake and scorpion bites were more common among farmers in the plantations. These findings are consistent with other Tanzanian studies that have documented similar patterns; for example, *Gilyoma and Chalya* noted that soft tissue injuries—particularly bruises and puncture wounds—were the most frequent presentations [1,2]. Based on these patterns, community-level training and public education should be considered, and the use of basic first-aid interventions and supplies should be actively promoted.

Management of animal-related injuries, including bites in Tanzania, is guided by the Standard Treatment Guidelines and Essential Medicines List of Tanzania (STG/NEMLIT) [14]. This guideline provides clear recommendations on clinical signs to monitor and detail both pharmacological and non-pharmacological interventions under a dedicated section on bites and stings. They emphasise early interventions such as vital sign stabilisation, immobilisation, pain management, and use of antibiotics, as well as tetanus toxoid and intravenous fluids where indicated. Our study found that while most clinicians adhered to the STG/NEMLIT, there are notable gaps, particularly concerning crocodile and marine animal bites, common in Mkinga but not explicitly addressed in the current guidelines. This suggests a need to expand future editions of the STG/NEMLIT to incorporate these local bite patterns.

Notably, using furosemide, a loop diuretic and a drug with no clinical indication for use in animal bites. The off-guideline medication, not included in STG/NEMLIT protocols for bite management, highlighting the need for continued clinical mentorship and reinforcement of treatment protocols. Furosemide can cause or exacerbate further complications in these patients, including dehydration and electrolyte abnormalities, making the bite victims worse, particularly those with systemic envenomation or anaphylaxis. We suspect, the inappropriate use may stem from diagnostic uncertainty or may be a misinterpretation of symptoms, for example, the clinician confusing the swelling or respiratory distress in animal injuries with a possible fluid overload due to their similarities. Poor adherence to guidelines has been documented elsewhere in Tanzania; a study from the Dodoma region found that only 29% of prescribers fully adhered to standard treatment guidelines, and incorrect medications were prescribed in 30% of cases [13]. These findings stress the need for ongoing medical education and targeted clinical mentorship, especially in rural settings, in order to promote safe, evidence-based care.

The integration of One Health strategies offers promising avenues for managing animal-related injuries. For example, a pilot of Integrated Bite Case Management (IBCM) across 20 Tanzanian districts resulted in a twofold increase in the detection of high-risk rabies exposures and improved timeliness of post-exposure prophylaxis (PEP), demonstrating the effectiveness of coordinated efforts between human and veterinary health sectors [24]. Additionally, studies among local communities, such as the *Sukuma t*ribe in *Busega* District, highlight traditional animal-based medicinal practices, emphasizing the importance of culturally sensitive approaches within clinical protocols to enhance acceptance and efficacy [25]. Building on these successes, it is imperative to revise national treatment guidelines (such as the STG/NEMLIT) to explicitly incorporate One Health algorithms for bite and envenomation management, ensuring a holistic, multisectoral approach. Such integration can facilitate earlier detection, reduce delays in care, prevent complications, and reduce mortality from animal-related injuries in Tanzania and similar resource-limited settings.

This study’s focus on lower-tier primary health facilities highlights the limited capacity of these settings to manage severe cases requiring advanced care. Common reasons for referral included the unavailability of antivenom, radiological imaging, and surgical services. These facilities typically lack access to diagnostic tools such as radiography and specialised laboratory tests, relying instead on basic assessments like random blood sugar tests, malaria rapid diagnostic tests, and haemoglobin levels. More advanced diagnostics, such as coagulation profiles or imaging for trauma, are only accessible at secondary or tertiary levels, necessitating frequent referrals. These findings depict the importance of an integrated care approach that ensures continuity and coordination across all levels of the health system. Even though the Tanzanian health system operates a decentralised model, rural PHCs often remain under-equipped. Equipping PHCs with essential emergency supplies, wound care kits, and training providers to stabilise and treat cases before referral could potentially yield better outcomes.

### Study limitations and generalizability

This study was conducted within a single district in Tanzania, which may limit the generalizability of its findings. However, unlike hospital-based studies, our community-based approach captures real-world treatment decisions and patient outcomes at the primary care level. In addition, Mkinga’s socioeconomic profile, which is characterised by fishing, pastoralism, and agriculture, is similar to many other rural districts in Tanzania, lending some broader relevance to the findings.

Limitations included incomplete clinical data, and the inconsistent record-keeping led to missing information on clinical presentations and diagnoses, which hindered comprehensive patient assessments and reduced the reliability of our epidemiological data. To mitigate this shortcoming, we conducted qualitative interviews with clinicians, which provided essential context and enriched our interpretation of the available quantitative information.

Another notable gap is the lack of diagnostic support; the absence of routine diagnostic tools such as radiography for detecting retained fangs or fractures, and laboratory assessments like coagulation profiles, limited our ability to definitively diagnose envenomation and accurately assess injury severity. Consequently, diagnoses were primarily clinical rather than confirmatory, raising the possibility of misclassification and underestimation of true cases.

Furthermore, our reliance on retrospective registry entries introduced potential documentation bias, as some clinically relevant details may have been inaccurately recorded or omitted. While this affects internal validity, the study still offers valuable insights into the management of animal-related injuries in resource-limited rural settings and highlights critical areas for improvement.

Lastly, qualitative interviews were conducted exclusively with facility in-charges (head clinicians). While thematic saturation was achieved, we acknowledge a potential bias arising from limiting respondents to this single cadre. Expanding the range of perspectives in future research by including other healthcare providers such as nurses, frontline clinicians, and traditional healers, would offer a more comprehensive understanding of care dynamics. Notably, our findings indicate that traditional healers are frequently consulted in rural settings, often serving as a parallel or complementary source of care. Thus, including these voices would provide richer insights into treatment pathways and inform the development of culturally grounded, co-created interventions tailored to rural health systems in management of animal related injuries.

### Implications and future directions

By acknowledging these limitations, we aim to transparently demonstrate how they may have influenced our findings and interpretations. Addressing these gaps in future studies is essential. Prospective, multi-regional or national-level research could improve both internal validity and generalizability across diverse socioeconomic and ecological contexts. Additionally, evaluating the effectiveness of community education initiatives and healthcare provider training programs remains a priority. We recommend that future studies be designed with larger, more representative samples to enable robust inferential analysis and risk stratification by animal type and clinical outcome.

## Conclusion and recommendations

Animal-related injuries range widely in severity, from mild pain to life-threatening complications. This study supports our hypothesis that the clinical management and outcomes of such injuries in rural Tanzania are significantly impacted by limited resources, inconsistent documentation, and variation in treatment practices. We observed notable gaps in care delivery, including the use of off-guideline medications, absence of antivenom, delayed referrals, and incomplete clinical records, reflecting systemic constraints and knowledge gaps at primary health care levels. Furthermore, the wide diversity of injuries, from dog bites to crocodile attacks, alongside delays in seeking care, contributed to complications such as amputations and, in some cases, death. These findings highlight the challenges of delivering standardized, evidence-based care in resource-limited rural settings, thereby validating our initial assumptions.

To address the identified gaps, we recommend a multifaceted approach tailored to rural primary health system needs. Strengthening diagnostic capacity at primary care facilities is critical and can be achieved by equipping providers with essential tools such as radiography and basic laboratory tests to assess envenomation and infections accurately. National treatment guidelines (STG/NEMLIT) should be updated by the Ministry of Health and medical associations to reflect the evolving epidemiology of animal injuries, including underrepresented exposures such as crocodile and marine bites. In-service training and structured mentorship programs are essential to enhance adherence to clinical protocols with emphasis on accurate medical documentation. It is also important to ensure reinforcement of referral systems from lower-level facilities, including the provision of well-equipped ambulances and trained personnel to ensure timely management of severe cases and appropriate standard transfer. At the community level, public health campaigns engaging local leaders, traditional healers, and community health workers should be implemented to promote early care-seeking and dispel misconceptions around traditional remedies. Future interventions should also be co-created to reflect the cultural realities of rural Tanzania, integrating both biomedical and traditional perspectives. Finally, we recommend the adoption of standardized electronic case reporting tools with required clinical fields to improve the quality, completeness, and utility of routine data for both patient care and health system planning.

## Supporting information

S1 File Qualitative data collection tool. This file presents the in-depth interview guide used to explore healthcare providers’ experiences and perspectives on the clinical presentation, management, and outcomes of animal-related injuries in rural Tanzania. The tool was administered to heads of health facilities in Mkinga District. It includes participant socio-demographic information, followed by a series of open-ended questions with probing prompts designed to elicit detailed responses on life-saving medications, clinical presentations, treatment approaches, outcomes, challenges, and recommendations for improving care for envenomation and animal bite cases.
(PDF)

S2 File SPSS output. This supplementary file presents detailed crosstabulations examining the relationships between facility level, patient disposition, nature of bites, and treatment practices in bites cases. The tables display counts and percentages of patients managed across dispensaries, and health centers, highlighting how admission, discharge, and referral rates vary by facility. Additionally, the data illustrate treatment patterns—including administration of tetanus toxoid, antibiotics, steroids, and antivenoms—across different types of bites such as snake, dog, insect, and arachnid. These analyses provide valuable insights into clinical management approaches tailored to bite type and healthcare setting, based on valid case records from the *Management_spss.sav* dataset.
(PDF)
