# Supplementary material for: From injury to outcome: A mixed-methods study of animal-related injuries in a rural district of Tanzania
Source: PLoS Negl Trop Dis. 2025 Sep 2;19(9):e0013494. doi: 10.1371/journal.pntd.0013494 (PMC12416828; doi:10.1371/journal.pntd.0013494)
Supplement: S1 File — This file presents the in-depth interview guide used to explore healthcare providers’ experiences and perspectives on the clinical presentation, management, and outcomes of animal-related injuries in rural Tanzania. The tool was administered to heads of health facilities in Mkinga District. It includes participant socio-demographic information, followed by a series of open-ended questions with probing prompts designed to elicit detailed responses on life-saving medications, clinical presentations, treatment approaches, outcomes, challenges, and recommendations for improving care for envenomation and animal bite cases. (PDF) [file pntd.0013494.s001.pdf]

Qualitative Data Collection Tool

Objective: To explore healthcare providers' experiences and perspectives on Clinical Presentation, Management and Outcome of Animal Related Injuries and in Rural Tanzania.

In-Depth Interview Guide for Heads of Health Facilities in Mkinga District

Participant Number: …………………………….

Table 1: Socio-Demographic Characteristics of the Study Participant

- **Sex: __________________________**
- **Age (years): _____________**
- **Highest Level of Professional Education: _______________________________________**
- **Level of the Health Facility: ___________________**
- **Years of Work Experience: ________________**

Interview Guide Questions and Probes

1. What is your understanding of life-saving medications used for insect and animal envenomation?

Probes:

- Which medications are considered life-saving in envenomation cases?
- What types of envenomation (e.g., snakebites, scorpion stings, spider bites, bee stings) are most common in your setting?
- Why is it important to have these medications readily available?
- What are the consequences if these medications are unavailable when needed?

2. Thank you for the response. Can you describe the typical clinical presentations of patients who come in with insect or animal envenomation?

Probes:

- What early signs and symptoms do you observe)?
- Are there specific symptoms associated with particular species or types of venom?
- How does severity vary between patients?
- What complications are commonly seen (probe. Respiratory distress, renal failure, paralysis, coagulopathy)?
- How do delays in care affect the clinical presentation?

3. What treatment approaches do you typically use for managing envenomation cases?

Probes:

- What specific medications or antidotes do you use (e.g., antivenoms, adrenaline, corticosteroids, antibiotics)?
- Is there a standard treatment protocol or clinical guideline that you follow?
- Do you use supportive therapies (e.g., fluids, pain control, respiratory support)? If so, when and how?
- How do you decide whether to refer a patient to a higher-level facility?
- Are all necessary medications and supplies usually available when needed?

4. In your experience, what are the typical outcomes of treatment for insect or animal envenomation cases?

Probes:

- Do most patients recover fully? If not, what kinds of complications or disabilities occur?
- Have you observed any deaths related to envenomation? What were the contributing factors?
- How does the timing of treatment affect outcomes?
- Are there specific groups (e.g., children, elderly, rural patients) who tend to have worse outcomes?

5. What are the main challenges you face in managing insect and animal envenomation cases?

Probes:

- Are there any shortages or stockouts of key medications (e.g., antivenoms)?
- Is staff adequately trained to recognize and treat envenomation?
- What challenges do you face in diagnosis or classification of envenomation?
- Are there delays in accessing emergency medications?
- How is the referral and transport system functioning for severe cases?

6. What would help improve the management and outcomes of envenomation cases in your facility or region?

Probes:

- What improvements are needed in medicine availability or supply chain?
- What training or capacity building would you recommend for healthcare staff?
- Are there guidelines or protocols you think should be developed or updated?
- What community-level interventions might help (e.g., awareness, first aid)?
