# Supplementary material for: From injury to outcome: A mixed-methods study of animal-related injuries in a rural district of Tanzania
Source: PLoS Negl Trop Dis. 2025 Sep 2;19(9):e0013494. doi: 10.1371/journal.pntd.0013494 (PMC12416828; doi:10.1371/journal.pntd.0013494)
Supplement: S2 File — This supplementary file presents detailed crosstabulations examining the relationships between facility level, patient disposition, nature of bites, and treatment practices in bites cases. The tables display counts and percentages of patients managed across dispensaries, and health centers, highlighting how admission, discharge, and referral rates vary by facility. Additionally, the data illustrate treatment patterns—including administration of tetanus toxoid, antibiotics, steroids, and antivenoms—across different types of bites such as snake, dog, insect, and arachnid. These analyses provide valuable insights into clinical management approaches tailored to bite type and healthcare setting, based on valid case records from the Management_spss.sav dataset. (PDF) [file pntd.0013494.s002.pdf]

SPSS OUTPUT

FILE='C:\Users\Windows 11\Desktop\VIHSM_Folder\Envenomation_second_manuscript\Management_spss.sav'.

DATASET NAME DataSet1 WINDOW=FRONT.

CROSSTABS /TABLES=Facilitylevel BY PatientDisposition /FORMAT=AVALUE TABLES /CELLS=COUNT /COUNT ROUND CELL.

Notes

Output Created		02-JUN-2025 12:50:38
Comments		
Input	Data	C:\Users\Windows 11\Desktop\VIHSM_Folder\Envenomation_second_manuscript\Management_spss.sav
	Active Dataset	DataSet1
	Filter	<none>
	Weight	<none>
	Split File	<none>
	N of Rows in Working Data File	998
Missing Value Handling	Definition of Missing	User-defined missing values are treated as missing.
	Cases Used	Statistics for each table are based on all the cases with valid data in the specified range(s) for all variables in each table.
Syntax		CROSSTABS /TABLES=Facilitylevel BY PatientDisposition /FORMAT=AVALUE TABLES /CELLS=COUNT /COUNT ROUND CELL.
Resources	Processor Time	00:00:00,00
	Elapsed Time	00:00:00,01

Dimensions Requested	2
Cells Available	349496

[

DataSet1] C:\Users\Windows 11\Desktop\VIHSM_Folder\Envenomation_second_manuscript\Management_spss.sav

Case Processing Summary

	Cases					
	Valid		Missing		Total	
	N	Percent	N	Percent	N	Percent
Facility level * Patient Disposition	998	100.0%	0	0.0%	998	100.0%

Facility level * Patient Disposition Crosstabulation

Count

		Patient Disposition				Total
			Admitted	Discharged	Referred	
Facility level		647	0	0	0	647
	Dispensary	0	0	257	19	276
	Health Centre	0	2	68	5	75
Total		647	2	325	24	998

Notes

Output Created		02-JUN-2025 12:51:41
Comments		
Input	Data	C:\Users\Windows 11\Desktop\VIHSM_Folder\Envenomation_second_manuscript\Management_spss.sav
	Active Dataset	DataSet1
	Filter	<none>
	Weight	<none>
	Split File	<none>
	N of Rows in Working Data File	998
Missing Value Handling	Definition of Missing	User-defined missing values are treated as missing.
	Cases Used	Statistics for each table are based on all the cases with valid data in the specified range(s) for all variables in each table.
Syntax		CROSSTABS<br>/TABLES=Facilitylevel BY PatientDisposition<br>/FORMAT=AVALUE TABLES<br>/CELLS=COUNT ROW<br>/COUNT ROUND CELL.
Resources	Processor Time	00:00:00,02
	Elapsed Time	00:00:00,01
	Dimensions Requested	2
	Cells Available	349496

Case Processing Summary

	Cases					
	Valid		Missing		Total	
	N	Percent	N	Percent	N	Percent
Facility level * Patient Disposition	998	100.0%	0	0.0%	998	100.0%

			Patient Disposition			
				Admitted	Discharged	Referred
Facility level		Count	647	0	0	0
		% within Facility level	100.0%	0.0%	0.0%	0.0%
	Dispensary	Count	0	0	257	19
		% within Facility level	0.0%	0.0%	93.1%	6.9%
	Health Centre	Count	0	2	68	5
		% within Facility level	0.0%	2.7%	90.7%	6.7%
Total		Count	647	2	325	24
		% within Facility level	64.8%	0.2%	32.6%	2.4%

Facility level * Patient Disposition Crosstabulation

			Total
Facility level		Count	647
		% within Facility level	100.0%
	Dispensary	Count	276
		% within Facility level	100.0%
	Health Centre	Count	75
		% within Facility level	100.0%
Total		Count	998
		% within Facility level	100.0%

```
CROSSTABS
  /TABLES=Natureofbites BY TetatusToxoid Antipain Antibiotics Adrenaline steroid ANTIHISTAMINE
    AntirabiesAnivenonAntidote Dressing
  /FORMAT=AVALUE TABLES
  /CELLS=COUNT ROW
  /COUNT ROUND CELL.
```

Types of Bite * Wound Care (Dressing and/or Cleaning) Crosstabulation

			Wound Care (Dressing and/or Cleaning)			Total
				2	1	
Types of Bite		Count	647	0	0	647
		% within Types of Bite	100.0%	0.0%	0.0%	100.0%
	Arachnids Bites (Spider, Scorpion)	Count	0	14	1	15
		% within Types of Bite	0.0%	93.3%	6.7%	100.0%
	Cat	Count	0	2	0	2
		% within Types of Bite	0.0%	100.0%	0.0%	100.0%
	Dog	Count	0	31	13	44
		% within Types of Bite	0.0%	70.5%	29.5%	100.0%
	Human being	Count	0	3	0	3
		% within Types of Bite	0.0%	100.0%	0.0%	100.0%
	Insect bite (wasp, bees)	Count	0	130	4	134
		% within Types of Bite	0.0%	97.0%	3.0%	100.0%
	Marine bite	Count	0	12	3	15
		% within Types of Bite	0.0%	80.0%	20.0%	100.0%
	Other animal related Injuries/Non Bite	Count	0	47	11	58
		% within Types of Bite	0.0%	81.0%	19.0%	100.0%
	Snake	Count	0	60	3	63
		% within Types of Bite	0.0%	95.2%	4.8%	100.0%
	Unidentified	Count	0	12	0	12
		% within Types of Bite	0.0%	100.0%	0.0%	100.0%
Total		Count	647	311	35	993
		% within Types of Bite	65.2%	31.3%	3.5%	100.0%

Types of Bite * Antirabies/Antivenom/Antidote Crosstabulation

			Antirabies/Antivenom/Antidote			Total
				2	1	
Types of Bite		Count	647	0	0	647
		% within Types of Bite	100.0%	0.0%	0.0%	100.0%
	Arachnids Bites (Spider, Scorpion)	Count	0	15	0	15
		% within Types of Bite	0.0%	100.0%	0.0%	100.0%
	Cat	Count	0	2	0	2
		% within Types of Bite	0.0%	100.0%	0.0%	100.0%
	Dog	Count	0	33	11	44
		% within Types of Bite	0.0%	75.0%	25.0%	100.0%
	Human being	Count	0	3	0	3
		% within Types of Bite	0.0%	100.0%	0.0%	100.0%
	Insect bite (wasp, bees)	Count	0	132	2	134
		% within Types of Bite	0.0%	98.5%	1.5%	100.0%
	Marine bite	Count	0	15	0	15
		% within Types of Bite	0.0%	100.0%	0.0%	100.0%
	Other animal related Injuries/Non Bite	Count	0	58	0	58
		% within Types of Bite	0.0%	100.0%	0.0%	100.0%
	Snake	Count	0	58	5	63
		% within Types of Bite	0.0%	92.1%	7.9%	100.0%
	Unidentified	Count	0	11	1	12
		% within Types of Bite	0.0%	91.7%	8.3%	100.0%
Total		Count	647	327	19	993
		% within Types of Bite	65.2%	32.9%	1.9%	100.0%

Types of Bite * Antihistamine Crosstabulation

			Antihistamine			Total
				2	1	
Types of Bite		Count	647	0	0	647
		% within Types of Bite	100.0%	0.0%	0.0%	100.0%
	Arachnids Bites (Spider, Scorpion)	Count	0	0	15	15
		% within Types of Bite	0.0%	0.0%	100.0%	100.0%
	Cat	Count	0	0	2	2
		% within Types of Bite	0.0%	0.0%	100.0%	100.0%
	Dog	Count	0	41	3	44
		% within Types of Bite	0.0%	93.2%	6.8%	100.0%
	Human being	Count	0	1	2	3
		% within Types of Bite	0.0%	33.3%	66.7%	100.0%
	Insect bite (wasp, bees)	Count	0	33	101	134
		% within Types of Bite	0.0%	24.6%	75.4%	100.0%
	Marine bite	Count	0	12	3	15
		% within Types of Bite	0.0%	80.0%	20.0%	100.0%
	Other animal related Injuries/Non Bite	Count	0	35	23	58
		% within Types of Bite	0.0%	60.3%	39.7%	100.0%
	Snake	Count	0	32	31	63
		% within Types of Bite	0.0%	50.8%	49.2%	100.0%
	Unidentified	Count	0	5	7	12
		% within Types of Bite	0.0%	41.7%	58.3%	100.0%
Total		Count	647	159	187	993
		% within Types of Bite	65.2%	16.0%	18.8%	100.0%

Types of Bite * Steroid Crosstabulation

			Steroid			Total
				2	1	
Types of Bite		Count	647	0	0	647
		% within Types of Bite	100.0%	0.0%	0.0%	100.0%
	Arachnids Bites (Spider, Scorpion)	Count	0	5	10	15
		% within Types of Bite	0.0%	33.3%	66.7%	100.0%
	Cat	Count	0	1	1	2
		% within Types of Bite	0.0%	50.0%	50.0%	100.0%
	Dog	Count	0	39	5	44
		% within Types of Bite	0.0%	88.6%	11.4%	100.0%
	Human being	Count	0	2	1	3
		% within Types of Bite	0.0%	66.7%	33.3%	100.0%
	Insect bite (wasp, bees)	Count	0	46	88	134
		% within Types of Bite	0.0%	34.3%	65.7%	100.0%
	Marine bite	Count	0	9	6	15
		% within Types of Bite	0.0%	60.0%	40.0%	100.0%
	Other animal related Injuries/Non-Bite	Count	0	34	24	58
		% within Types of Bite	0.0%	58.6%	41.4%	100.0%
	Snake	Count	0	13	50	63
		% within Types of Bite	0.0%	20.6%	79.4%	100.0%
	Unidentified	Count	0	6	6	12
		% within Types of Bite	0.0%	50.0%	50.0%	100.0%
Total		Count	647	155	191	993
		% within Types of Bite	65.2%	15.6%	19.2%	100.0%

Types of Bite * Adrenaline Crosstabulation

			Adrenaline			Total
				2	1	
Types of Bite		Count	647	0	0	647
		% within Types of Bite	100.0%	0.0%	0.0%	100.0%
	Arachnids Bites (Spider, Scorpion)	Count	0	12	3	15
		% within Types of Bite	0.0%	80.0%	20.0%	100.0%
	Cat	Count	0	2	0	2
		% within Types of Bite	0.0%	100.0%	0.0%	100.0%
	Dog	Count	0	42	2	44
		% within Types of Bite	0.0%	95.5%	4.5%	100.0%
	Human being	Count	0	3	0	3
		% within Types of Bite	0.0%	100.0%	0.0%	100.0%
	Insect bite (wasp, bees)	Count	0	121	12	133
		% within Types of Bite	0.0%	91.0%	9.0%	100.0%
	Marine bite	Count	0	12	3	15
		% within Types of Bite	0.0%	80.0%	20.0%	100.0%
	Other animal related Injuries/Non-Bite	Count	0	54	4	58
		% within Types of Bite	0.0%	93.1%	6.9%	100.0%
	Snake	Count	0	55	7	62
		% within Types of Bite	0.0%	88.7%	11.3%	100.0%
	Unidentified	Count	0	12	0	12
		% within Types of Bite	0.0%	100.0%	0.0%	100.0%
Total		Count	647	313	31	991
		% within Types of Bite	65.3%	31.6%	3.1%	100.0%

Types of Bite * Antibiotics Crosstabulation

			Antibiotics			Total
				2	1	
Types of Bite		Count	647	0	0	647
		% within Types of Bite	100.0%	0.0%	0.0%	100.0%
	Arachnids Bites (Spider, Scorpion)	Count	0	13	2	15
		% within Types of Bite	0.0%	86.7%	13.3%	100.0%
	Cat	Count	0	1	1	2
		% within Types of Bite	0.0%	50.0%	50.0%	100.0%
	Dog	Count	0	20	24	44
		% within Types of Bite	0.0%	45.5%	54.5%	100.0%
	Human being	Count	0	0	3	3
		% within Types of Bite	0.0%	0.0%	100.0%	100.0%
	Insect bite (wasp, bees)	Count	0	107	27	134
		% within Types of Bite	0.0%	79.9%	20.1%	100.0%
	Marine bite	Count	0	9	6	15
		% within Types of Bite	0.0%	60.0%	40.0%	100.0%
	Other animal related Injuries/Non-Bite	Count	0	35	23	58
		% within Types of Bite	0.0%	60.3%	39.7%	100.0%
	Snake	Count	0	46	17	63
		% within Types of Bite	0.0%	73.0%	27.0%	100.0%
	Unidentified	Count	0	10	2	12
		% within Types of Bite	0.0%	83.3%	16.7%	100.0%
Total		Count	647	241	105	993
		% within Types of Bite	65.2%	24.3%	10.6%	100.0%

Types of Bite * Antipain Crosstabulation

			Antipain			Total
				2	1	
Types of Bite		Count	647	0	0	647
		% within Types of Bite	100.0%	0.0%	0.0%	100.0%
	Arachnids Bites (Spider, Scorpion)	Count	0	7	8	15
		% within Types of Bite	0.0%	46.7%	53.3%	100.0%
	Cat	Count	0	2	0	2
		% within Types of Bite	0.0%	100.0%	0.0%	100.0%
	Dog	Count	0	25	19	44
		% within Types of Bite	0.0%	56.8%	43.2%	100.0%
	Human being	Count	0	1	2	3
		% within Types of Bite	0.0%	33.3%	66.7%	100.0%
	Insect bite (wasp, bees)	Count	0	57	77	134
		% within Types of Bite	0.0%	42.5%	57.5%	100.0%
	Marine bite	Count	0	3	12	15
		% within Types of Bite	0.0%	20.0%	80.0%	100.0%
	Other animal related Injuries/Non-Bite	Count	0	29	29	58
		% within Types of Bite	0.0%	50.0%	50.0%	100.0%
	Snake	Count	0	42	20	62
		% within Types of Bite	0.0%	67.7%	32.3%	100.0%
	Unidentified	Count	0	7	5	12
		% within Types of Bite	0.0%	58.3%	41.7%	100.0%
Total		Count	647	173	172	992
		% within Types of Bite	65.2%	17.4%	17.3%	100.0%

Types of Bite * Tetanus Toxoid Crosstabulation

			Tetatus Toxoid			Total
				2	1	
Types of Bite		Count	647	0	0	647
		% within Types of Bite	100.0%	0.0%	0.0%	100.0%
	Arachnids Bites (Spider, Scorpion)	Count	0	13	2	15
		% within Types of Bite	0.0%	86.7%	13.3%	100.0%
	Cat	Count	0	2	0	2
		% within Types of Bite	0.0%	100.0%	0.0%	100.0%
	Dog	Count	0	13	31	44
		% within Types of Bite	0.0%	29.5%	70.5%	100.0%
	Human being	Count	0	2	1	3
		% within Types of Bite	0.0%	66.7%	33.3%	100.0%
	Insect bite (wasp, bees)	Count	0	130	4	134
		% within Types of Bite	0.0%	97.0%	3.0%	100.0%
	Marine bite	Count	0	11	4	15
		% within Types of Bite	0.0%	73.3%	26.7%	100.0%
	Other animal related Injuries/Non-Bite	Count	0	34	24	58
		% within Types of Bite	0.0%	58.6%	41.4%	100.0%
	Snake	Count	0	52	11	63
		% within Types of Bite	0.0%	82.5%	17.5%	100.0%
	Unidentified	Count	0	11	1	12
		% within Types of Bite	0.0%	91.7%	8.3%	100.0%
Total		Count	647	268	78	993
		% within Types of Bite	65.2%	27.0%	7.9%	100.0%
